# Myofibrosarcoma of the maxillary sinus with orbital floor involvement: a case report

**DOI:** 10.1093/jscr/rjag920

**Published:** 2026-10-14

**Authors:** Adam El Hassouni, Rajae Borki, Aymene Benmansour, Omar Rami, Ilham Rkain, Hicham Mimouni

**Affiliations:** Department of Otolaryngology—Head and Neck Surgery, Mohammed VI University Hospital, Route de Rabat Km 17, BP 398, Gzenaya, Tangier 90000, Morocco; Department of Otolaryngology—Head and Neck Surgery, Mohammed VI University Hospital, Route de Rabat Km 17, BP 398, Gzenaya, Tangier 90000, Morocco; Faculty of Medicine and Pharmacy of Tangier, Abdelmalek Essaâdi University, Route de Rabat Km 15, BP 365, Gzenaya, Tangier 90000, Morocco; Department of Anatomy, Abdelmalek Essaâdi University, Route de Rabat Km 15, BP 365, Gzenaya, Tangier 90000, Morocco; Department of Otolaryngology—Head and Neck Surgery, Mohammed VI University Hospital, Route de Rabat Km 17, BP 398, Gzenaya, Tangier 90000, Morocco; Department of Otolaryngology—Head and Neck Surgery, Mohammed VI University Hospital, Route de Rabat Km 17, BP 398, Gzenaya, Tangier 90000, Morocco; Department of Otolaryngology—Head and Neck Surgery, Mohammed VI University Hospital, Route de Rabat Km 17, BP 398, Gzenaya, Tangier 90000, Morocco; Faculty of Medicine and Pharmacy of Tangier, Abdelmalek Essaâdi University, Route de Rabat Km 15, BP 365, Gzenaya, Tangier 90000, Morocco; Department of Otolaryngology—Head and Neck Surgery, Mohammed VI University Hospital, Route de Rabat Km 17, BP 398, Gzenaya, Tangier 90000, Morocco; Faculty of Medicine and Pharmacy of Tangier, Abdelmalek Essaâdi University, Route de Rabat Km 15, BP 365, Gzenaya, Tangier 90000, Morocco

**Keywords:** myofibrosarcoma, maxillary sinus, sinonasal sarcoma, orbital floor, radiotherapy

## Abstract

Myofibrosarcoma is a rare spindle-cell malignancy with myofibroblastic differentiation, and primary maxillary sinus involvement is exceptional. A 36-year-old woman presented with progressive unilateral nasal obstruction, purulent rhinorrhea, and hemifacial pain. Computed tomography and magnetic resonance imaging demonstrated a destructive 44 × 45 × 57 mm right maxillary sinus mass involving the orbital floor and extending into the nasal cavity, ethmoid cells, and pterygopalatine fossa. Histology and immunohistochemistry supported the diagnosis of myofibrosarcoma. Partial maxillectomy with titanium orbital floor reconstruction was performed. Several soft-tissue margins were positive, whereas gingival and bony margins were clear. Postoperative radiotherapy delivered 66 Gy in 33 fractions. This case illustrates the diagnostic complexity of sinonasal spindle-cell tumors and the individualized role of radiotherapy when local control is threatened by anatomically constrained positive-margin disease.

## Introduction

Myofibrosarcoma is a rare malignant mesenchymal tumor composed of spindle cells showing myofibroblastic differentiation [1–3]. The World Health Organization classification specifically recognizes low-grade myofibroblastic sarcoma [1, 4]; this terminology is not used interchangeably here with the definitive histological diagnosis of myofibrosarcoma in the present case. Primary nasal cavity and paranasal sinus involvement is uncommon, and maxillary sinus cases are limited to isolated reports and small series [5–12]. We report a locally advanced right maxillary sinus myofibrosarcoma with orbital floor involvement, positive soft-tissue margins, and postoperative radiotherapy.

## Case report

A 36-year-old woman with no relevant medical history presented with 3 months of progressive right-sided nasal obstruction, purulent rhinorrhea, and hemifacial pain. Nasal endoscopy showed a tumor occupying the right nasal cavity, without clinically evident cervical lymphadenopathy.

Contrast-enhanced computed tomography (CT) demonstrated a 44 × 45 × 57 mm heterogeneous mass centered on the right maxillary sinus. It eroded the sinus walls and orbital floor, extended into the nasal cavity and ethmoid cells, filled the pterygopalatine fossa, and destroyed the superior maxillary alveolar bone (Fig. 2). Magnetic resonance imaging (MRI) confirmed extension toward the premaxillary tissues, buccal fat pad, pterygoid region, zygomatic bone, and ethmoid cells, with displacement of the orbital floor and inferior rectus muscle but no definite deep intraconal or intracranial invasion (Fig. 3).

Endoscopic biopsy showed a malignant fascicular spindle-cell proliferation with moderate atypia and a slightly myxoid stroma. Tumor cells expressed smooth muscle actin and calponin, with focal h-caldesmon expression, and were negative for the pancytokeratin AE1/AE3 antibody cocktail, desmin, S100, cluster of differentiation 34 (CD34), and signal transducer and activator of transcription 6 (STAT6). The Ki-67 index was ~40%, indicating high proliferative activity. The histomorphological and immunohistochemical findings supported the diagnosis of myofibrosarcoma (Fig. 4).

Following multidisciplinary review, partial maxillectomy was performed. The orbital floor was reconstructed with a titanium plate, allowing orbital preservation (Fig. 1). Definitive histology confirmed a 5-cm myofibrosarcoma. The superior, posterior, lateral, and fibrous soft-tissue margins were involved, while gingival and bony margins were clear.

**Figure 1:**
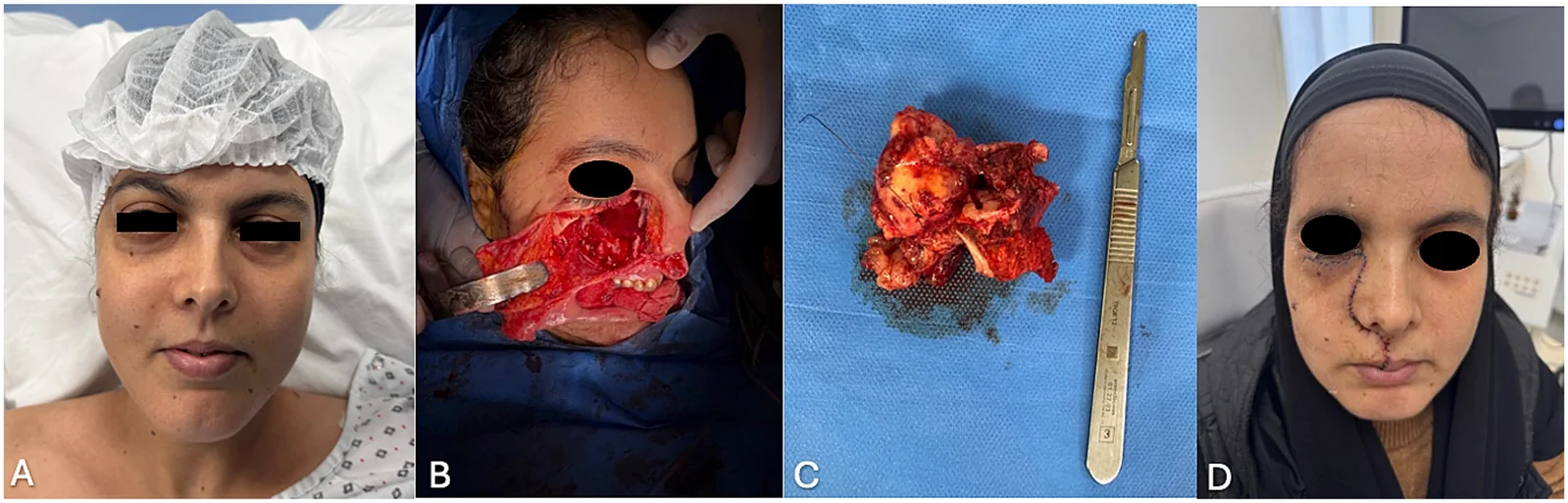
Clinical, intraoperative, and postoperative findings; (A) preoperative frontal view showing right midfacial swelling; (B) intraoperative view during partial maxillectomy; (C) gross surgical specimen after tumor resection; (D) postoperative appearance following partial maxillectomy and orbital floor reconstruction.

**Figure 2:**
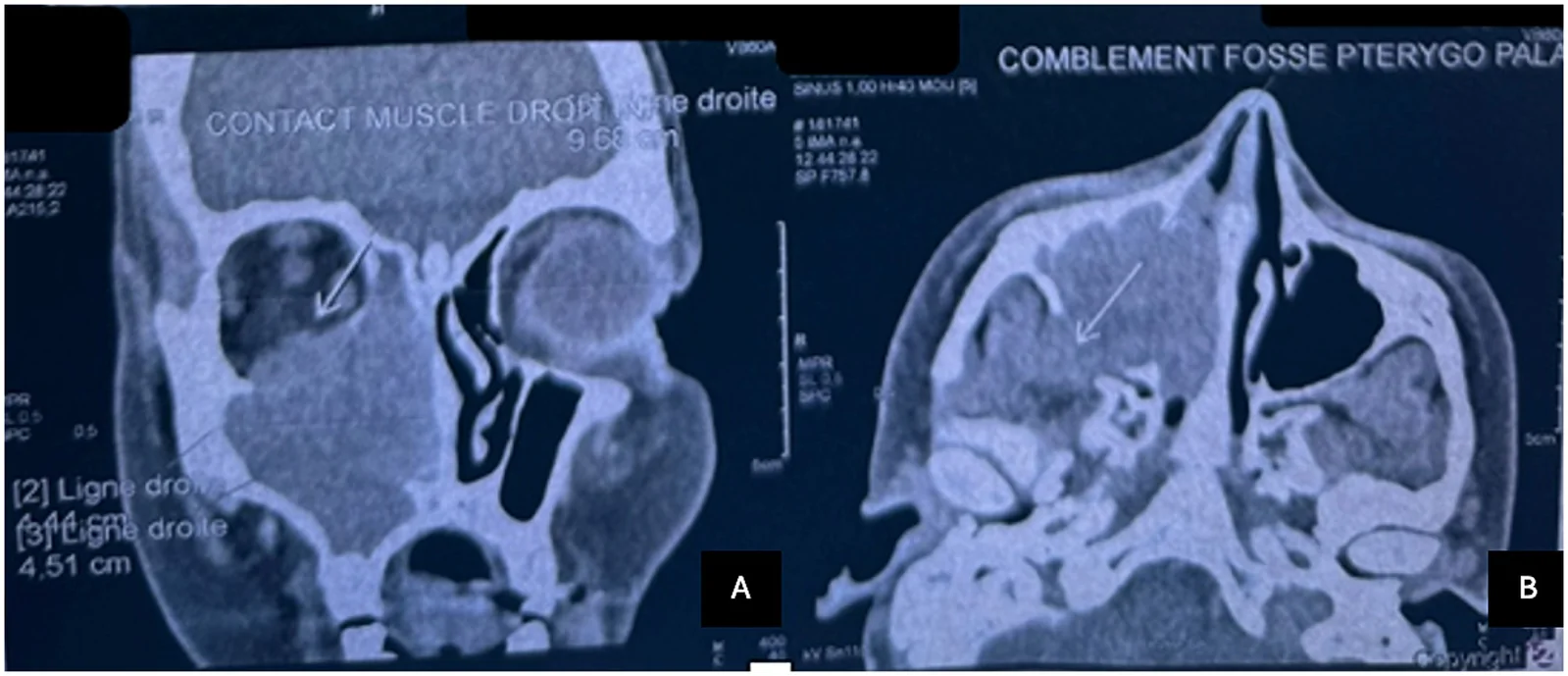
Contrast-enhanced CT of the paranasal sinuses; (A) coronal image showing a destructive right maxillary sinus mass with orbital floor erosion and superior extension toward the orbit; (B) axial image demonstrating extension into the right nasal cavity and pterygopalatine fossa; arrows indicate the tumor and areas of extension.

**Figure 3:**
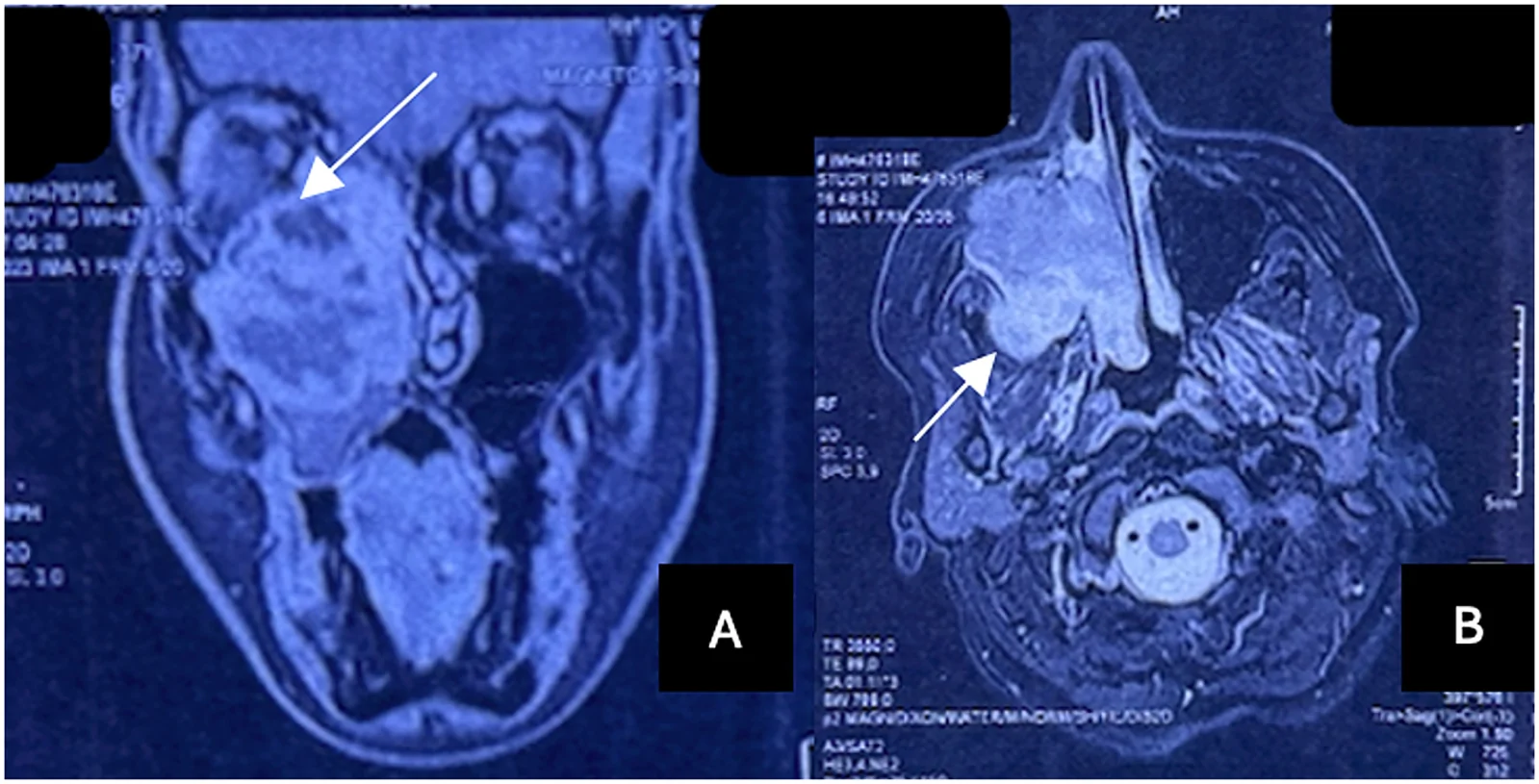
MRI of the right maxillary sinus tumor; (A) coronal image demonstrating superior extension toward the orbital floor; (B) axial image showing the tumor and its relationship with adjacent deep facial spaces; arrows indicate the lesion.

**Figure 4:**
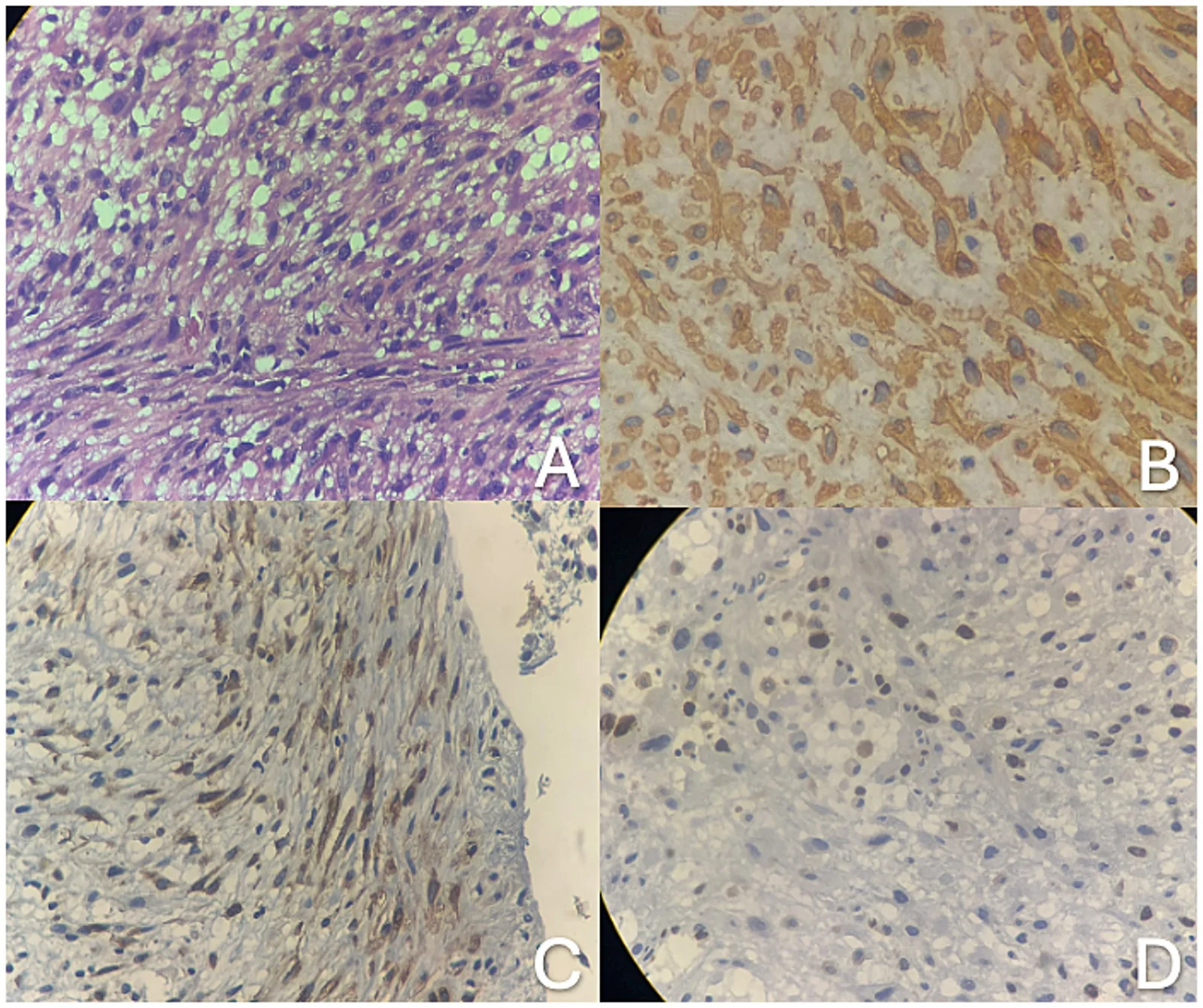
Histopathological and immunohistochemical findings; (A) hematoxylin and eosin staining (original magnification ×40) showing fascicles of atypical spindle cells in a slightly myxoid stroma; (B) smooth muscle actin positivity; (C) calponin positivity; (D) Ki-67 nuclear labeling in ~40% of tumor cells.

Given the locally advanced site, orbital floor involvement, high proliferative index, and positive soft-tissue margins, postoperative radiotherapy delivered 66 Gy in 33 fractions. A transient decrease in right visual acuity resolved within 1 month. At 6 months, the patient was clinically well without evident local recurrence; continued clinical and radiological surveillance is ongoing.

## Discussion

Sinonasal myofibrosarcoma is diagnostically challenging because spindle-cell carcinoma, leiomyosarcoma, malignant peripheral nerve sheath tumor, melanoma, solitary fibrous tumor, inflammatory myofibroblastic tumor, and biphenotypic sinonasal sarcoma may show overlapping morphology [13]. In this case, the combined expression profile supported myofibroblastic differentiation, while negative epithelial, neural, and fibroblastic markers argued against major alternatives. Molecular testing was unavailable, limiting complete exclusion of molecularly defined mimics.

Surgery with negative margins remains the treatment cornerstone. However, the orbit, skull base, palate, pterygopalatine fossa, infraorbital nerve, and masticator space frequently restrict wide sinonasal resection. Published maxillary and sinonasal cases demonstrate heterogeneous treatment and outcomes (Table 1). In our patient, partial maxillectomy achieved macroscopic removal and orbital preservation but left multiple positive soft-tissue margins.

**Table 1:** Clinicopathological features of reported maxillary sinus and sinonasal myofibrosarcoma cases

**Author, year**	**Age/sex**	**Site/side**	**Reported grade**	**Size**	**Presentation**	**Treatment**	**Outcome**
Bisceglia *et al*., 2001 [7]	24/M	Left maxilla	Low-grade	4 cm	Pain and midface swelling	Surgery; details not reported	No recurrence; follow-up 40 months
Meng *et al*., 2007, case 1 [6]	33/F	Left sinonasal region	Intermediate	6.5 cm	Nasal obstruction and rhinorrhea	Wide excision by Caldwell–Luc approach + RT 60 Gy	Died of disease; follow-up 12 months
Meng *et al*., 2007, case 6 [6]	28/F	Left sinonasal region	Intermediate	4 cm	Nasal obstruction and rhinorrhea	Wide excision by Caldwell–Luc approach	Local recurrence at ~6 months; follow-up 21 months
Gangwar *et al*., 2015 [8]	27/M	Maxilla	Reported as myofibrosarcoma/LGMS	6 × 5 cm	Not reported	Total maxillectomy + RT	No recurrence; follow-up 24 months
Gomez-Oliveira *et al*., 2015 [9]	75/F	Left maxillary sinus	Low-grade	Not reported	Pain, swelling, destructive mass	Maxillectomy + orbital floor reconstruction + free flap + adjuvant RT	Distant metastasis reported at ~1 year
Ghosh *et al*., 2019 [10]	35/M	Right maxillary sinus	Low-grade	Not reported	Exophthalmos	Extended medial maxillectomy ± chemotherapy, as reported	Local recurrence at ~6 years
Mydlak *et al*., 2024, case 1 [11]	46/M	Right maxillary sinus	Low-grade	8 cm	Pain, midface swelling, nasal obstruction	Expanded maxillectomy + reconstruction + IMRT 66 Gy	No recurrence; follow-up 15–18 months
Mydlak *et al*., 2024, case 2 [11]	45/M	Right maxillary sinus	Low-grade	8 cm	Exophthalmos, diplopia, visual impairment	Expanded surgery + adjuvant RT; later palliative chemotherapy	Recurrence and death; follow-up 26 months
Dabas *et al*., 2024 [12]	39/M	Left maxilla	Myofibrosarcoma	4.8 × 1.7 × 4.2 cm	Epistaxis, nasal obstruction, visual loss	Maxillectomy + reconstruction + adjuvant RT	Disease-free at 3 years
Present case	36/F	Right maxillary sinus	Descriptively intermediate-grade	5 cm on pathology	Hemifacial pain, nasal obstruction, nasal cavity mass	Partial maxillectomy + orbital floor reconstruction + postoperative RT 66 Gy/33 fractions	Transient visual acuity decrease with full recovery; favorable 6-month clinical follow-up

Abbreviations: F, female; Gy, gray; IMRT, intensity-modulated radiotherapy; LGMS, low-grade myofibroblastic sarcoma; M, male; RT, radiotherapy.

This table provides a comparative summary of the clinicopathological features, treatments, and outcomes of previously reported maxillary sinus and sinonasal myofibrosarcoma cases.

Terminology and histological grading are reported as described in the original publications.

Most available evidence regarding radiotherapy concerns low-grade myofibroblastic sarcoma rather than myofibrosarcoma. Population data in low-grade myofibroblastic sarcoma have not shown a clear survival benefit in unselected patients, but available studies incompletely account for margin status and local anatomical constraints [5, 14, 15]. These data should therefore be interpreted cautiously when extrapolated to the present case. Radiotherapy may be considered when local control is threatened by positive margins, recurrence, locally advanced disease, or a constrained head and neck location. The principal limitations of this report are the absence of molecular testing and short follow-up.

This case emphasizes the need for radiological, pathological, and surgical correlation in destructive maxillary sinus spindle-cell tumors. Complete excision should be pursued, while postoperative radiotherapy may be individualized for high-risk positive-margin disease. Long-term surveillance is essential.

## Reporting guideline

This case report was prepared in accordance with the CAse REport (CARE) guidelines.

## Supplementary Material

CARE_Checklist_rjag920

## Data Availability

Clinical data are not publicly available because of patient confidentiality but may be available from the corresponding author upon reasonable request and subject to ethical and legal restrictions.
