# Supplementary material for: Myofibrosarcoma of the maxillary sinus with orbital floor involvement: a case report
Source: J Surg Case Rep. 2026 Oct 14;2026(10):rjag920. doi: 10.1093/jscr/rjag920 (PMC13668423; doi:10.1093/jscr/rjag920)
Supplement: Supplementary file 1 [file care_checklist_rjag920.docx]

CARE Checklist – Completed for Submission

Manuscript title: Myofibrosarcoma of the maxillary sinus with orbital floor involvement: a case report

| CARE item | How addressed | Location |
| --- | --- | --- |
| 1. Title | Diagnosis and “case report” identified in the title. | Title page |
| 2. Keywords | Six specific keywords supplied. | Title page / Abstract page |
| 3. Abstract | Presentation, diagnosis, intervention, outcome, and main lesson summarized. | Abstract |
| 4. Introduction | Rarity, diagnostic difficulty, and reason for reporting explained. | Introduction |
| 5. Patient information | Age, sex, symptoms, duration, and relevant history described. | Case presentation |
| 6. Clinical findings | Nasal endoscopy and cervical findings described. | Case presentation |
| 7. Timeline | Presentation, surgery, radiotherapy, recovery, and follow-up reported chronologically. | Case presentation |
| 8. Diagnostic assessment | CT, MRI, morphology, immunohistochemistry, differential diagnosis, and limitations described. | Case presentation / Discussion |
| 9. Therapeutic intervention | Surgery, reconstruction, and radiotherapy dose described. | Case presentation |
| 10. Follow-up and outcomes | Visual toxicity, recovery, and six-month status described. | Case presentation |
| 11. Discussion | Literature context, reasoning, treatment rationale, and limitations discussed. | Discussion |
| 12. Patient perspective | Not available. | Not reported |
| 13. Informed consent | Written consent for publication and images obtained and retained. | Statements and Declarations |
